# Reduction of Neuroinflammation as a Common Mechanism of Action of Anorexigenic and Orexigenic Peptide Analogues in the Triple Transgenic Mouse Model of Alzheimer´s Disease

**DOI:** 10.1007/s11481-025-10174-w

**Published:** 2025-02-11

**Authors:** Anna Mengr, Zuzana Šmotková, Andrea Pačesová, Blanka Železná, Jaroslav Kuneš, Lenka Maletínská

**Affiliations:** 1 Institute of Organic Chemistry and Biochemistry of the Czech Academy of Sciences, Flemingovo Nám. 2, 160 00 Prague, Czech Republic; 2 First Faculty of Medicine, Charles University, Kateřinská 32, 12108 Prague, Czech Republic; 3 Institute of Physiology of the Czech Academy of Sciences, Vídeňská 1083, 142 00 Prague, Czech Republic

**Keywords:** Alzheimer’s disease, Neuroinflammation, 3xTg-AD mice, Orexigenic peptide analogues, Anorexigenic peptide analogues

## Abstract

Alzheimer’s disease (AD) is the most common form of dementia. Characterized by progressive neurodegeneration, AD typically begins with mild cognitive decline escalating to severe impairment in communication and responsiveness. It primarily affects cerebral regions responsible for cognition, memory, and language processing, significantly impeding the functional independence of patients. With nearly 50 million dementia cases worldwide, a number expected to triple by 2050, the need for effective treatments is more urgent than ever. Recent insights into the association between obesity, type 2 diabetes mellitus, and neurodegenerative disorders have led to the development of promising treatments involving antidiabetic and anti-obesity agents. One such novel promising candidate for addressing AD pathology is a lipidized analogue of anorexigenic peptide called prolactin-releasing peptide (palm^11^-PrRP31). Interestingly, anorexigenic and orexigenic peptides have opposite effects on food intake regulation, however, both types exhibit neuroprotective properties. Recent studies have also identified ghrelin, an orexigenic peptide, as a potential neuroprotective agent. Hence, we employed both anorexigenic and orexigenic compounds to investigate the common mechanisms underpinning their neuroprotective effects in a triple transgenic mouse model of AD (3xTg-AD mouse model) combining amyloid-beta (Aβ) pathology and Tau pathology, two hallmarks of AD. We treated 3xTg-AD mice for 4 months with two stable lipidized anorexigenic peptide analogues – palm^11^-PrRP31, and liraglutide, a glucagon-like peptide 1 (GLP-1) analogue – as well as Dpr^3^-ghrelin, a stable analogue of the orexigenic peptide ghrelin, and using the method of immunohistochemistry and western blot demonstrate the effects of these compounds on the development of AD-like pathology in the brain. Palm^11^-PrRP31, Dpr^3^-ghrelin, and liraglutide reduced intraneuronal deposits of Aβ plaque load in the hippocampi and amygdalae of 3xTg-AD mice. Palm^11^-PrRP31 and Dpr^3^-ghrelin reduced microgliosis in the hippocampi, amygdalae, and cortices of 3xTg-AD mice. Palm^11^-PrRP31 and liraglutide reduced astrocytosis in the amygdalae of 3xTg-AD mice. We propose that these peptides are involved in reducing inflammation, a common mechanism underlying their therapeutic effects. This is the first study to demonstrate improvements in AD pathology following the administration of both orexigenic and anorexigenic compounds, highlighting the therapeutic potential of food intake-regulating peptides in neurodegenerative disorders.

**Graphical Abstract:**

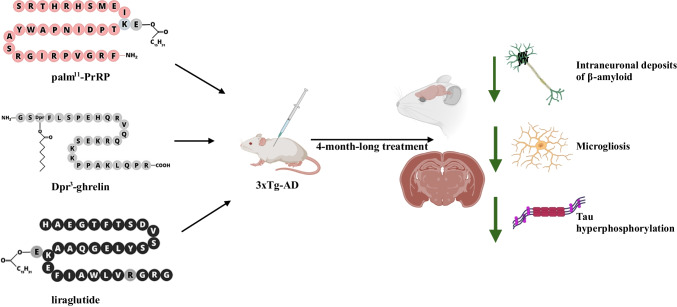

**Supplementary Information:**

The online version contains supplementary material available at 10.1007/s11481-025-10174-w.

## Background

Alzheimer’s disease (AD), the most common form of dementia, manifests as a progressive neurodegenerative condition. Initially characterized by mild cognitive decline, AD later progresses to severe impairment in verbal communication and environmental responsiveness. Pathologically, it primarily affects cerebral areas governing cognitive functions, memory encoding, and linguistic processing. Consequently, it profoundly impedes the functional independence of individuals and their ability to perform routine activities. Globally, there are nearly 50 million cases of dementia, a number projected to triple by 2050 (Lane et al. 2018).

The recently established connection between obesity and its related comorbidities, such as type 2 diabetes mellitus (T2DM), and development of neurodegenerative disorders has unveiled potential novel treatment avenues, namely antidiabetic and anti-obesity substances (Maletinska et al. 2019). Both T2DM, obesity, and AD share common molecular pathways, particularly insulin resistance and disrupted insulin signaling (Pomytkin et al. 2018). Insulin resistance, characteristic of both T2DM and obesity (Liu et al. 2016, 2011), impairs glucose metabolism and induces neuronal dysfunction, contributing to AD progression (Flores-(Flores-Cordero et al. 2022). Additionally, oxidative stress (Zhao & Zhao 2013) and neuroinflammation (Heneka et al. 2015), which are present in both T2DM and AD, exacerbate these pathological processes. Disturbances in the regulation of energy balance and neuropeptide metabolism, which are involved in food intake control, are also common to both AD and metabolic disorders (López-Gambero et al. 2021). In neurodegenerative diseases like AD, neurodegeneration is closely associated with aging, insulin and leptin resistance (Flores-Cordero et al. 2022; Liu et al. 2011), inflammation driven by cytokines (Kacířová et al. 2020), disruptions in energy balance regulation (López-Gambero et al. 2021) and more. These features also overlap with conditions such as T2DM and metabolic syndrome. Consequently, these disorders may be managed with similar, or potentially overlapping, therapeutic strategies. Therefore, drugs originally developed for obesity treatment (Li et al. 2015) could also hold promise for AD management, highlighting the essential role of neuropeptides in regulating neuronal function.

Subsequently, several modified analogues of anorexigenic peptides, which decrease food intake, like prolactin-releasing peptide (PrRP) or glucagon-like peptide 1 (GLP-1), showed promise in alleviating neuropathological changes in the brain connected to AD, such as improved spatial memory, reduced amyloid-beta (Aβ) plaques, Tau pathology and neuroinflammation, and at the same time increased neurogenesis and synaptogenesis in different preclinical rodent models od AD-like pathology (Holubová et al. 2019; Pacesova et al. 2022; Spolcova et al. 2015).

PrRP and its lipidized analogue palm^11^-PrRP31 have demonstrated multifaceted therapeutic potential in addressing AD pathology (Holubová et al. 2019; Mengr et al. 2021). For instance, in APP/PS1 mouse model a notable reduction in Aβ plaque accumulation in the hippocampus, along with decreased hippocampal microgliosis and cortical astrocytosis after treatment with palm^11^-PrRP31 was reported (Holubová et al. 2019). Additionally, palm^11^-PrRP31 displayed a tendency to enhance neurogenesis in the hippocampus while significantly decreasing Tau phosphorylation at [Thr231], one of the first epitopes phosphorylated in AD (Holubová et al. 2019). Furthermore, in THY-Tau22 mice characterized by mutated human Tau protein overexpression, palm^11^-PrRP31 not only improved short-term spatial memory, but also attenuated abnormal Tau phosphorylation and increased synaptic plasticity markers (Popelová, Pražienková, et al. 2018a, b). These findings suggest that palm^11^-PrRP31 and related analogues hold promise as potential therapeutic interventions for AD (Holubová et al. 2019; Mengr et al. 2021; Popelová, Pražienková, et al. 2018a, b; Spolcova et al. 2015).

Similarly, anti-obesity and antidiabetic drug liraglutide, a GLP-1 analogue, reduced Tau phosphorylation and enhanced memory and learning in various models exhibiting AD pathology (Holubová et al. 2019; McClean & Holscher 2014; McClean et al. 2015; Spolcova et al. 2015). Intriguingly, Paladugu et al. (Paladugu et al. 2021) showed that the intraperitoneal injection of liraglutide significantly mitigated neuroinflammatory responses in mice exhibiting both sporadic AD induced by the administration of streptozotocin (an insulin-cell toxin) into the lateral ventricles, as well as in 5xFAD mouse model exhibiting the Aβ pathology that represents familial form of AD, resulting in notable cognitive improvement. These results suggest that liraglutide exhibits neuroprotective effects and could serve as a prophylactic therapy of anti-inflammatory and anti-amyloid potential during the prodromal stages of AD (Paladugu et al. 2021). In another study involving APP/PS1 mice, liraglutide reduced Aβ plaque load and attenuated neuroinflammation (Holubová et al. 2019). However, studies involving patients with AD have indicated only modest positive effect (Edison et al. 2023; Hölscher 2024), liraglutide significantly slowed the progression of cognitive impairments, as evidenced by MRI scans showing less shrinkage in brain temporal and parietal lobe volumes, as well as in total grey matter cortical volume, compared to the placebo group. This indicates that liraglutide may help reduce neuronal loss in the brain (Edison et al. 2023, 2021). Semaglutide (Ozempic, Rybelsus), a novel GLP-1 agonist currently undergoing two phase-3 clinical trials in AD patients (NCT04777396 and NCT04777409), has demonstrated positive effects on AD pathology in a 3xTg-AD mouse model, including improved cognition and glucose metabolism (Wang et al. 2023).

Orexigenic peptides, which stimulate food intake, are the physiological counterparts of anorexigenic peptides. Many of them are produced in the brain, such as neuropeptide Y or agouti-related peptide (Schwartz et al. 2000). However, in the periphery, the known orexigenic peptide is ghrelin that is produced in the stomach (Kojima et al. 1999). Interestingly, despite the opposite effects of anorexigenic and orexigenic peptides on the regulation of food intake, both types are understood to be neuroprotective (Strnadova et al. 2024). However, the final mechanism of action remains unclear.

In our recent in vitro study, Popelova et al. (Popelová et al. 2018a) demonstrated that Dpr^3^-ghrelin, a stable ghrelin analogue, protected SH-SY5Y cells (a human-derived neuroblastoma cell line) against methylglyoxal-induced toxicity and apoptosis, indicating its potential utility as a treatment of neurodegenerative disorders. The authors also propose that the mechanism responsible for the antiapoptotic effect of Dpr^3^-ghrelin is mediated by the PKB/Akt and mitogen-activated protein kinases/extracellular signal-regulated kinase pathways (Popelová et al. 2018a). Several in vivo studies have identified ghrelin as a potential therapeutic agent in various neurological disorders, including AD, Parkinson’s disease, stroke, epilepsy, multiple sclerosis, and spinal cord injuries (Jeong et al. 2018; Miao et al. 2007; Moon et al. 2011). Evidence of the direct effect of ghrelin on AD-like pathology was first documented in the Senescence-Accelerated Mouse Prone 8 (SAMP8) model, widely used to examine the pathology of early AD defects (Diano et al. 2006; Pacesova et al. 2022). In agreement, improvements in the retention of T-maze foot-shock avoidance after ghrelin administration in 12- and 14-month-old mice were reported (Diano et al. 2006). Moon et al. (Moon et al. 2011) showed that systemic injections of ghrelin reduced microgliosis and prevented a neuronal loss caused by intrahippocampal administration of oligomeric forms of the Aβ peptide. Ghrelin was also proved to restore cognitive impairments, possibly through increased synaptogenesis (Moon et al. 2011). In another study, 5xFAD mice were treated with MK-0677, a nonpeptide agonist of ghrelin receptor GHS-R1a. The results showed that activation of the ghrelin receptor with MK-0677 inhibited Aβ burden, neuroinflammation, as well as neuronal and synaptic loss in the deep cortical layers (Jeong et al. 2018).

The frequently used 3xTg-AD effectively encompasses the key pathological features of AD (Oddo et al. 2003). These mice carry the human mutated genes APPSWE and PSEN1dE9, which induce the formation of Aβ plaques, as well as the human Tau P301L transgene, which leads to the formation of neurofibrillary tangles (Ramsden et al. 2005). These transgenes are primarily expressed within the central nervous system (CNS), notably in the hippocampus and cerebral cortex. We employed this AD mouse model to explore the common neuroprotective mechanisms of the anorexigenic and orexigenic peptides, namely palm^11^-PrRP31 and liraglutide as well as Dpr^3^-ghrelin. To the best of our knowledge, this is the first study comparing both anorexigenic and orexigenic hormones within a single model.

## Methods

### Peptide Synthesis

Palm^11^-PrRP31 (SRTHRHSMEIK (N-γ-E(N-palmitoyl)) TPDINPAWYASRGIRPVGRF-NH_2_) and Dpr^3^-ghrelin (GS Dpr (N-octanoyl)FLSPEHQKAQQRKESKKPPAKLQPR) were synthesized and purified at the Institute of Organic Chemistry and Biochemistry of the Czech Academy of Sciences (IOCB CAS) as described previously (Maletinska et al. 2012; Pražienková et al. 2017). Liraglutide (Victoza, Novo Nordisk A/S, Bagsværd, Denmark) was obtained from a pharmacy.

### Animals

Animal experiments adhered to the ethical guidelines outlined in Act No. 246/1992 of the Czech Republic and were approved by the Committee for Experiments with Laboratory Animals of the Czech Academy of Sciences. 3xTg-AD mice hemizygous for Tg (APPswe, PSEN1dE9, and Tau P301L) were obtained from the Jackson Laboratory (Bar Harbor, Maine, USA). As wild-type (WT) controls mice of B6129SF2/J – 101045 origin were recommended by Jackson laboratory. All mice used in this study were males. The mice were kept at an IOCB CAS animal facility in groups of five per cage. They were given unrestricted access to water and a standard rodent chow diet Ssniff® R/M-H (ssniff Spezialdiäten GmbH, Soest, Germany) containing 33, 9, and 58% of calories from protein, fat, and carbohydrates, respectively. The mice were housed under controlled conditions at a constant temperature of 23 ± 2 ℃ and on a 12-h light/dark cycle (lights on at 05:00). Body weight (BW) was monitored on a weekly basis for the duration of the experiment.

Peptides were administered from the age of six months and lasted four months. 3xTg-AD mice were treated once daily (except weekends) with saline, palm^11^-PrRP31, Dpr^3^-ghrelin, or liraglutide. We selected the doses of palm^11^-PrRP31 (5 mg/kg administered subcutaneously (SC) once daily) and liraglutide (0.2 mg/kg SC once daily) based on our prior research (Holubová et al. 2019; Spolcova et al. 2015).

The central effects of peripherally administered palm^11^-PrRP31 (5 mg/kg) were confirmed by a significant increase in c-Fos immunoreactivity in the hypothalamic arcuate nucleus, the paraventricular nucleus and dorsomedial nucleus, which are involved in the regulation of food intake (Pražienková et al. 2017). The reduction of Aβ plaque load in hippocampus in APP/PS1 mice after palm^11^-PrRP31 treatment was observed as well as significantly reduced hippocampal microgliosis (Holubova et al. 2018).

Liraglutide was used as a comparator in our previous studies (Holubová et al. 2019; Spolcova et al. 2015) where the dose of 0.2 mg/kg/day reduced the Aβ plaque load in the hippocampus of APP/PS1 mice (Holubova et al. 2018). Liraglutide was also used by Zhang et al. (Zhang et al. 2020) where liraglutide improved the cognitive function of diabetic mice after SC injections of liraglutide (0. 240 mg/kg/day).

The dose of Dpr^3^-ghrelin (5 mg/kg administered SC once daily) was established based on our recent studies demonstrating its impact on food intake in fed mice (Holubova et al. 2018). To date, no study has explored the neuroprotective effect of Dpr^3^-ghrelin. All compounds were dissolved in saline. WT mice were treated once daily (except weekends) with saline.

At the age of 10 months, blood samples were obtained from the tail veins of mice after overnight fasting for the determination of insulin and glucose. Blood ethylenediaminetetraacetic acid (EDTA) plasma was then isolated and preserved at –80 °C. Subsequently, the mice were deeply anesthetized with pentobarbital (50 mg/kg, intraperitoneally, Sigma-Aldrich, St. Louis, MO, USA), and the left heart ventricle was punctured, blood was collected, and EDTA plasma was isolated and stored at − 80 °C. Mice were transcardially perfused using ice-cold saline supplemented with heparin (10 U/ml, Zentiva, Prague, Czech Republic) White (subcutaneous and epididymal) and brown adipose tissue, the liver and brain were dissected and weighed. The brains were maintained on ice to prevent tissue degradation. The right hemisphere of the brain used for immunohistochemistry (IHC) was placed in 4% paraformaldehyde dissolved in 0.1 M phosphate-buffered saline (PBS) at a pH of 7.4 for 24 h and subsequently stored at 4 °C in 30% sucrose in 0.1 M PBS at a pH of 7.4 with 0.01% sodium azide until sectioning. The hippocampus of the left hemisphere was dissected, flash-frozen in dry ice immediately after dissection and stored at − 80 °C until homogenization.

### Determination of Biochemical Parameters

Blood glucose levels were determined using a glucometer (ARKRAY, Tokyo, Japan). C-reactive protein (CRP) levels in blood plasma were determined using a CRP ELISA kit (Thermo Scientific, Frederick, MD, USA). Plasma insulin concentrations were assessed using a RIA kit (Merck Millipore, Burlington, MA, USA) and plasma leptin concentrations using an ELISA kit (Merck Millipore, Burlington, MA, USA).

### Western Blotting

Hippocampi were processed, and Western blotting (WB) was performed as previously described (Holubová et al. 2019). The following primary antibodies were diluted 1: 1000 in casein blocking buffer with 0.01% Tween-20: glycogen-synthase kinase 3β (GSK-3β), pGSK-3β [Ser9], IκB kinase β (IKKβ, Cell Signaling Technology, Danvers, Massachusetts, USA), pIKK α/β [Ser176/180] (Cell Signaling Technology, Danvers, Massachusetts, USA), nuclear factor κB (NFκB, Cell Signaling Technology, Danvers, Massachusetts, USA), protein phosphatase 2A subunit C (PP2A subC) (Cell Signaling Technology, Danvers, Massachusetts, USA), stress-activated protein kinase/Jun-amino-terminal kinase (SAPK/JNK, Cell Signaling Technology, Danvers, Massachusetts, USA), pSAPK/JNK [Thr183/Tyr185] (Cell Signaling Technology, Danvers, Massachusetts, USA), Tau5, pTau [Ser396] (Invitrogen, Waltham, MA USA), Tau1 (Merck Millipore, Darmstadt, Germany), Toll-like receptor 4 (TLR-4, Cell Signaling Technology, Danvers, Massachusetts, USA), nuclear and β-actin (Sigma-Aldrich, Saint-Louis, Missouri, USA). The following secondary antibodies were used: IRDye 680RD Goat anti-Rabbit IgG Secondary Antibody, or IRDye 800CW Goat anti-Mouse IgG Secondary Antibody (both from LI-COR Biosciences, NE, USA). Fluorescence was visualized using the LI-COR Odyssey CLx Imager (LI-COR Biosciences, NE, USA). The data obtained were quantified in Empiria Studio Software (LI-COR Biosciences, NE, USA).

### Brain Immunohistochemistry

**Fig. 1:**
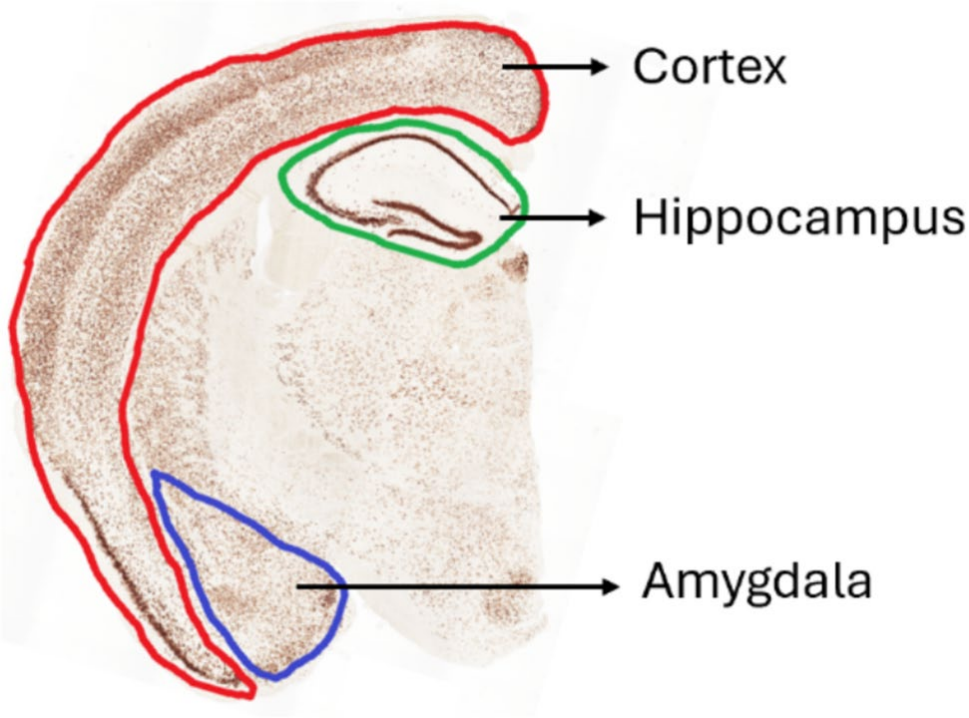
The representative figure of analysed areas in immunohistochemical evaluation of 3xTg-AD mouse brains: hippocampus (green), cortex (red) and amygdala (blue)

Paraformaldehyde-fixed right brain hemispheres were rapidly frozen. Coronal sections of 30-μm thickness were cut using a Leica cryostat (Leica Biosystems, Nussloch, Germany). The initial section was selected randomly, followed by sampling of every twelfth section thereafter. A detailed description of the chromogenic staining protocol with 3,3′-diaminobenzidine (DAB) solution (Vector Laboratories, Burlingame, CA, USA) is described in our previous study (Holubová et al. 2019). The dilution of primary antibodies for 6E10, ionized calcium binding adaptor molecule 1 (Iba1), and glial fibrillary acidic protein (GFAP) is shown in Table 1. The stained sections were observed and captured using the Olympus IX83 inverted optical microscope (Tokyo, Japan) using cellSens Imaging software (Olympus, Tokyo, Japan). Images encompassing the entire area of interest were captured at 10 × magnification. The percentage of the stained area was subsequently quantified using ImageJ software (NIH, Bethesda, MD, USA). We manually selected the area of interest (shown in Fig. 1) in accordance with the mouse brain atlas featured in Paxinos and Franklin’s the Mouse Brain in Stereotaxic Coordinates (Franklin & Paxinos 2008). The findings are presented as a percentage relative to the saline-treated 3xTg-AD control group to facilitate comparisons across the different staining series.

**Table 1:** List of primary antibodies and their appropriate dilution ratios for brain immunohistochemistry

Antibody (Ab)	Manufacturer and cat. no	Dilution	Specificity
**6E10** rabbit chimeric monoclonal Ab (mAb)	Invitrogen/Thermo Fisher Scientific, Waltham, MA, USA cat. no. 71–5800	1:800	Binds to amino acid residues 1–16 of Aβ
**Iba1** rabbit mAb	Wako, Osaka, Japan cat. no. 019–19741	1:2000	Specific to microglia and macrophages, but not cross-reactive with neurons and astrocytes
**GFAP** rabbit polyclonal Ab (pAb)	Invitrogen/Thermo Fisher Scientific, Waltham, MA, USA cat. no. PA5-16,291	1:400	Heavily and specifically expressed in astrocytes and certain astroglia of the central nervous system
**AT-100** mouse mAb	Invitrogen/Thermo Fisher Scientific, Waltham, MA, USA cat. no. MN1060	1:200	Recognizes hyperphosphorylated Tau in paired helical filaments (PHF) and detects an abnormal phosphorylation site unique to PHF-Tau; its epitope contains phosphorylated Ser214 and phosphorylated Thr212 residues
**Tau5** mouse mAb	Invitrogen/Thermo Fisher Scientific, Waltham, MA, USA cat. no. AHB0042	1:200	Recognizes total Tau protein; mostly found in the axons of neurons and the cytosol; associated with plasma membrane components

In preparation for fluorescent immunohistochemistry, the sample preparation mirrored the aforementioned procedure, except for the omission of H_2_O_2_ incubation. Permeabilization of brain slices was performed in 0.2% Triton X-100 in 1 × Tris-buffered saline (TBS). We then performed a blocking step with M.O.M blocking serum (Vector Laboratories, Inc., Burlingame, CA, USA) to minimize nonspecific antibody binding (60 min at room temperature (RT)) followed by a two-day incubation with the primary antibody at 4 °C (the respective dilutions are given in Table 1). Brain sections were exposed to primary antibodies against pTau AT-100 and total Tau5. After three washes with 0.2% Triton X-100 in TBS, sections were treated with anti-mouse AlexaFluor 488 conjugate (Thermo Fisher Scientific, Waltham, MA, USA) at a 1:1000 dilution for 90 min at RT. Next, whole neurons (1:100, 30 min at RT) were stained with NeuroTrace 435/455 Blue Fluorescent Nissl Stain (Thermo Fisher Scientific, Waltham, MA, USA). The stained sections were observed and captured using the Zeiss LSM 980 confocal microscope with Airyscan2 using a 40 × water objective. Schematic images were designed using scientific image and illustration software from BioRender.

### Statistical Analysis

Data are presented as the mean ± standard error of the mean (SEM). Statistical analysis was performed using GraphPad Prism 9 Software (San Diego, CA, USA), including one-way ANOVA followed by Dunnett’s multiple-comparison test (for IHC, WB and measurement of CRP) or two-way ANOVA followed by Bonferroni´s multiple comparison test (for BW, Week cumulative food intake and OGTT), as indicated in Figure legends. The significance level was set at *p* < 0.05.

## Results

### Palm^11^-PrRP31, Dpr^3^-ghrelin, or Liraglutide did not Influence Morphometric and Metabolic Parameters

**Fig. 2:**
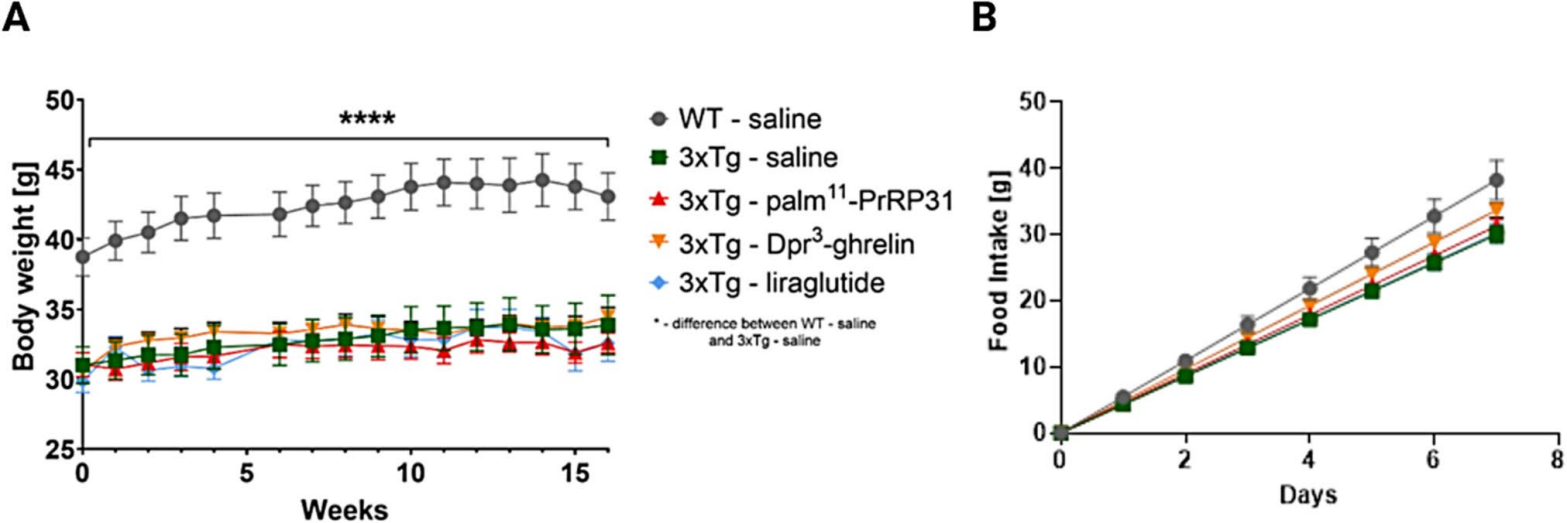
**A-B** Body weight (**A**) and Week cumulative food intake (**B**). Data are presented as the mean ± SEM. A two-way ANOVA was used to analyze differences between groups over time. Significance levels are indicated as follows: *p < 0.05, ****p < 0.0001 (*n* = 7–10 mice per group)

Nor anorexigenic analogues palm^11^-PrRP31 and liraglutide, neither orexigenic analogue Dpr^3^-ghrelin influence the BW of 3xTg-AD mice during the 4-month-long treatment (Fig. 2.A). The only significant difference in the BW from the beginning of the treatment throughout the experiment was observed between WT and 3xTg-AD mice treated with saline. The same results were obtained for cumulative food intake (Fig. 2.B). We observed significant increase in the level of leptin (*p* < 0.0001) and non-significant increase in the level of insulin between saline-treated WT group compared to the saline-treated 3xTg-AD group (Suppl. Figure 1.). However, no analogue significantly affected the level of these hormones (Suppl. Figure 1.A–C).

We observed significantly elevated CRP protein levels in the saline-treated 3xTg-AD group (*p* = 0.05) compared to the saline-treated WT group. The four-month treatment with palm^11^-PrRP31, Dpr^3^-ghrelin, or liraglutide tended to decrease CRP protein levels (Suppl. Figure 4.).

### Palm^11^-PrRP31, Dpr^3^-ghrelin, and Liraglutide Reduced Intraneuronal Aβ Plaque-load Deposits in the Hippocampi and Amygdalae of 3xTg-AD Mice but not in Cortices

**Fig. 3:**
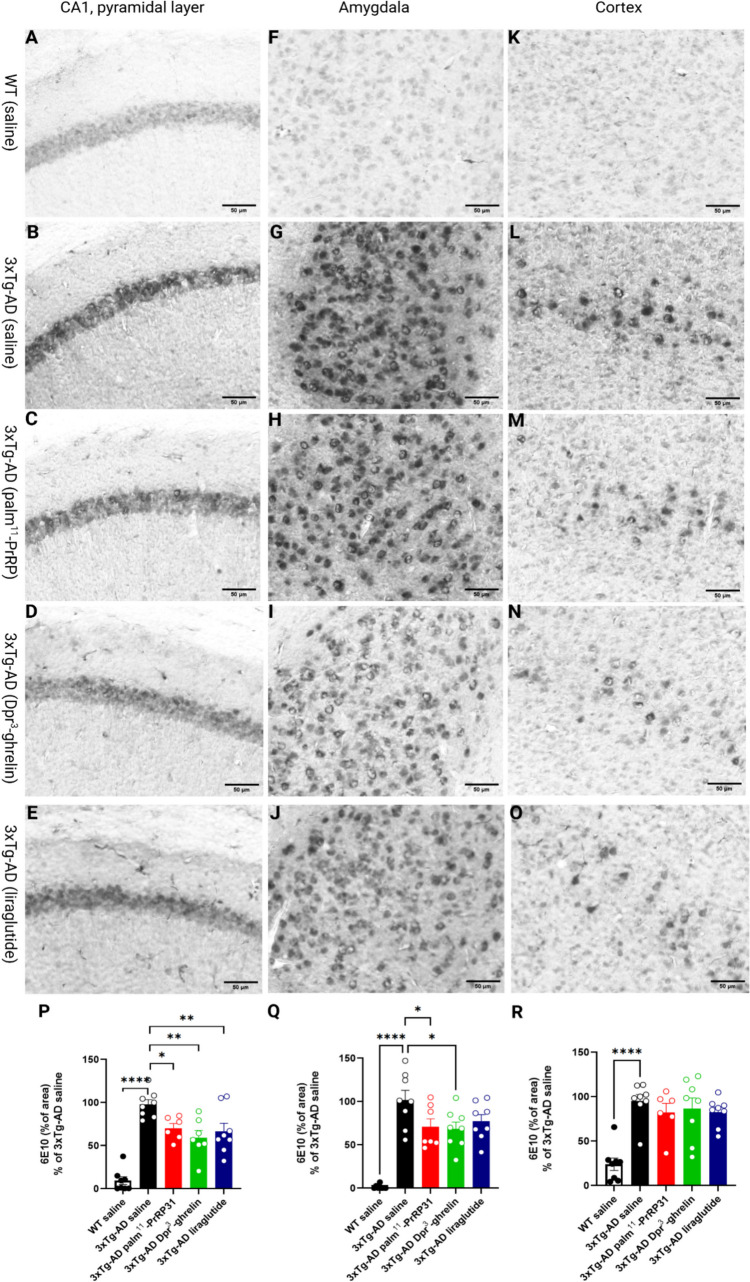
Reduction of intraneuronal Aβ deposits in the hippocampi, and amygdalae of 3xTg-AD mice after treatment with palm^11^-PrRP31, Dpr^3^-ghrelin, or liraglutide. Representative microscopic images of 3xTg-AD mice treated with saline, palm^11^-PrRP31, Dpr.^3^-ghrelin, or liraglutide and their age-matched WT controls immunohistochemically stained for intraneuronal Aβ (immunostained with 6E10 antibody) in the (**A**) hippocampus, (**F**) amygdala, and (**K**) cortex. Quantification of immunohistochemical staining of intraneuronal Aβ in the (**P**) hippocampus, (**Q**) amygdala, and (**R**) cortex. Stained area is expressed as a percentage of the saline-treated 3xTg-AD group to enable the comparison of multiple staining series. Data are presented as the mean ± SEM. A one-way ANOVA with Dunnett’s test was used to analyze differences between groups. Significance levels are indicated as follows: *p < 0.05, **p < 0.01, ***p < 0.001, ****p < 0.0001 (*n* = 8–9 mice per group)

Immunohistochemical staining of the brain sections of 3xTg-AD mice showed development of intraneuronal Aβ plaques (immunodetected with 6E10 antibody) in the hippocampi (*p* < 0.0001), amygdalae (*p* < 0.0001), and cortices (*p* < 0.0001) of 10-month-old mice compared to the saline-treated WT group. While the four months of treatment with our analogues did not influence the level of Aβ plaques in the cortex, palm^11^-PrRP31 (*p* = 0.04), Dpr^3^-ghrelin (*p* = 0.002), or liraglutide (*p* = 0.009) significantly reduced the number of intraneuronal Aβ plaques in the hippocampus (Fig. 3.P). Moreover, palm^11^-PrRP31 (*p* = 0.04) and Dpr^3^-ghrelin (*p* = 0.02) significantly reduced the number of intraneuronal Aβ plaques in the amygdala; however, the effect of liraglutide was not significant (*p* = 0.12) (Fig. 3.Q).

### Palm^11^-PrRP31 and Dpr^3^-ghrelin Reduced Microgliosis in the Hippocampi, Amygdalae, and Cortices of 3xTg-AD Mice

**Fig. 4:**
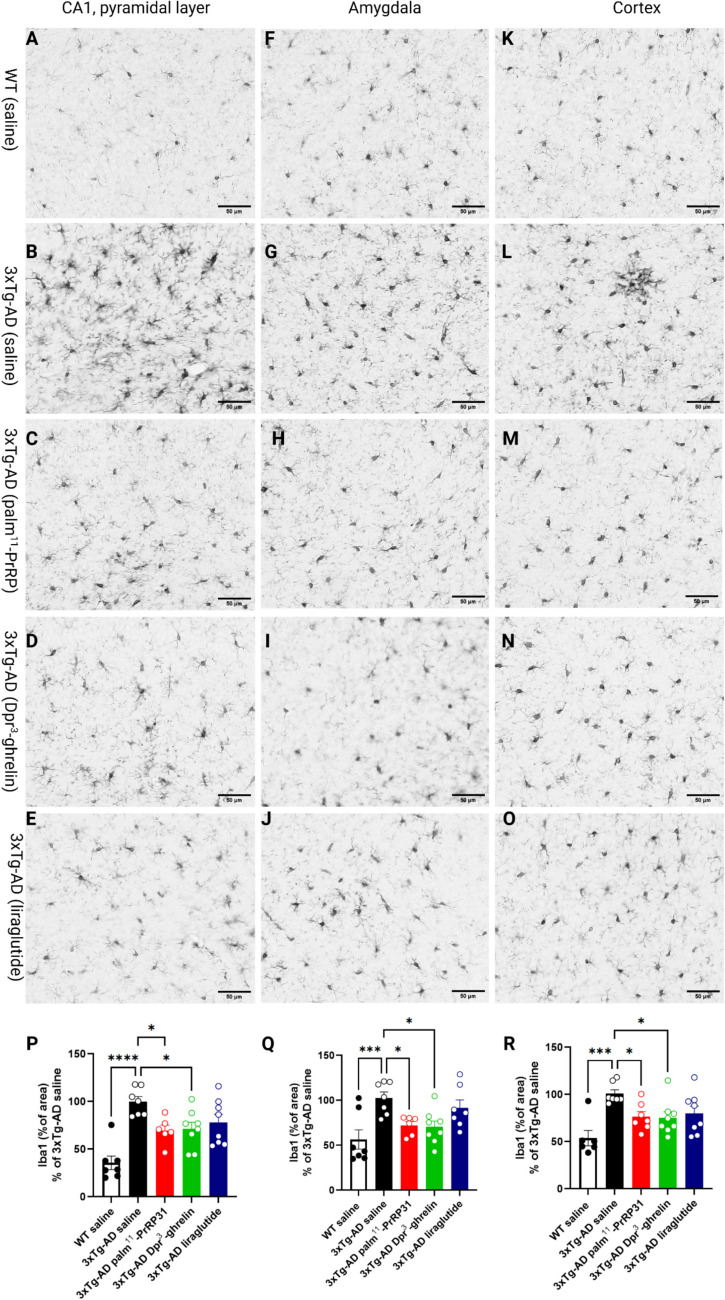
Reduction of microgliosis in the hippocampi, amygdalae, and cortices of 3xTg-AD mice after treatment with palm^11^-PrRP31, Dpr^3^-ghrelin, or liraglutide. Representative microscopic images of 3xTg-AD mice treated with (**B**,** G**,** L**) saline, (**C**,** H**,** M**) palm^11^-PrRP31, (**D**,** I**,** N**) Dpr.^3^-ghrelin, or (**E**,** J**,** O**) liraglutide and their (**A**,** F**,** K**) age-matched WT controls. Images show immunohistochemical staining for microglial marker Iba1. (**P**–**R**) Quantification of immunohistochemical staining for Iba1 in the (**P**) hippocampus, (**Q**) amygdala, and (**R**) cortex. The stained area is expressed as a percentage of the saline-treated 3xTg-AD group to enable the comparison of multiple staining series. Data are presented as the mean ± SEM. A one-way ANOVA with Dunnett’s test was used to analyze differences between groups. Significance levels are indicated as follows: *p < 0.05, ***p < 0.001, ****p < 0.0001 (*n* = 8–9 mice per group)

Immunohistochemical staining for Iba1, a marker of both activated and resting microglia and macrophages, revealed an increased number of activated microglia in the hippocampi (*p* < 0.0001), amygdalae (*p* < 0.001), and cortices (*p* = 0.0002) of 10-month-old saline-treated 3xTg-AD mice compared to the saline-treated WT group. Activated microglia typically display distinct morphological changes characterized by reduced ramification, fewer branches, and a more pronounced hypertrophic state compared to non-activated ramified microglia. Four months of treatment with palm^11^-PrRP31 (*p* = 0.03) and Dpr^3^-ghrelin (*p* = 0.02) significantly reduced microgliosis in the hippocampi of 3xTg-AD mice, but the effect of liraglutide was not significant (Fig. 4.P). Similarly, treatment with Dpr^3^-ghrelin (*p* = 0.02) and palm^11^-PrRP31 (*p* = 0.05) significantly reduced microgliosis in the amygdalae of 3xTg-AD mice, but the effect of liraglutide was not significant (Fig. 4.Q). Four months of treatment with palm^11^-PrRP31 (*p* = 0.05) and Dpr^3^-ghrelin (*p* = 0.03) significantly reduced microgliosis in the cortices of 3xTg-AD mice. However, the effect of liraglutide (*p* = 0.09) was not significant, as quantified by the percentage of the immunohistochemically stained area.

### Palm^11^-PrRP31 and Liraglutide Reduced Astrocytosis in the Amygdalae of 3xTg-AD Mice

**Fig. 5:**
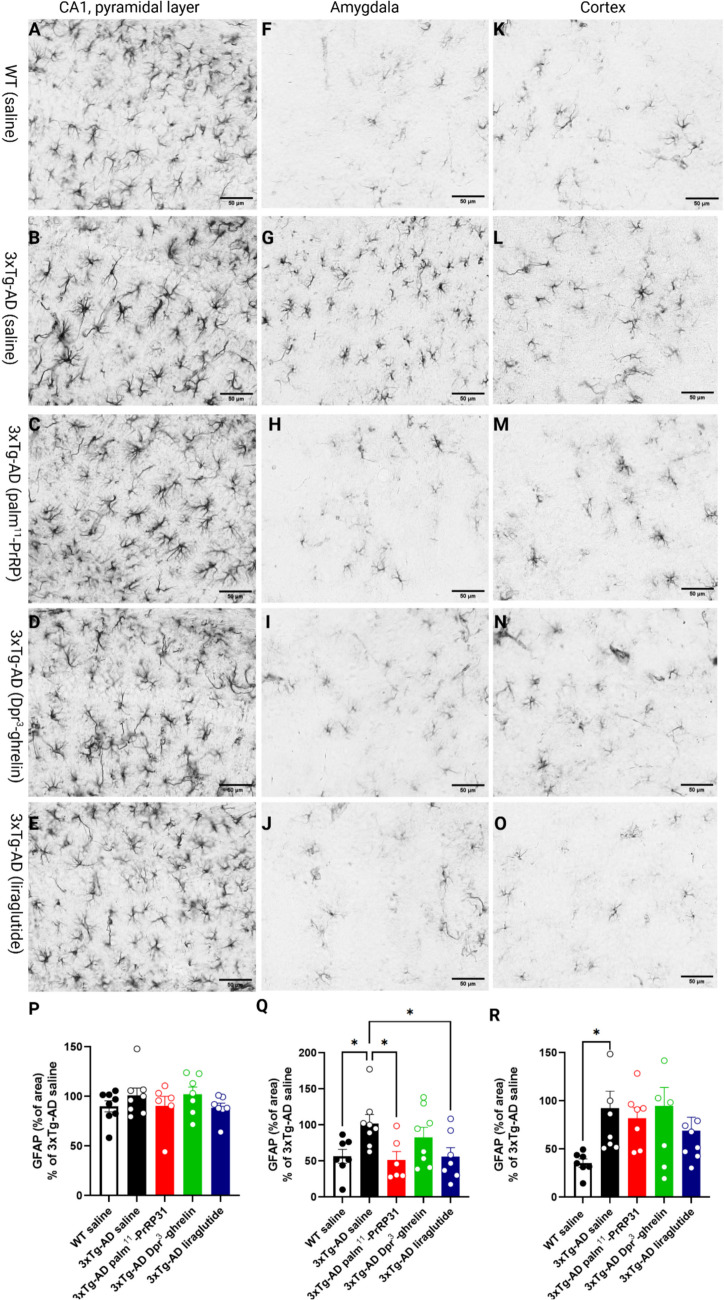
Reduction of astrocytosis in the amygdalae of 3xTg-AD mice after treatment with palm^11^-PrRP31, or liraglutide. Representative microscopic images of 3xTg-AD mice treated with (**B**,** G**,** L**) saline, (**C**,** H**,** M**) palm^11^-PrRP31, (**D**,** I**,** N**) Dpr^3^-ghrelin, or (**E**,** J**,** O**) liraglutide and their (**A**, **F**, **K**) age-matched WT controls. Images show immunohistochemical staining for glial fibrillary acidic protein (GFAP). (**P**–**R**) Quantification of immunohistochemical staining of GFAP in the (**P**) hippocampus, (**Q**) amygdala, and (**R**) cortex. The stained area is expressed as a percentage of the saline-treated 3xTg-AD group to enable the comparison of multiple staining series. Data are presented as the mean ± SEM. A one-way ANOVA with Dunnett’s test was used to analyze differences between groups. Significance levels are indicated as follows: *p < 0.05 (*n* = 8 or 9 mice per group)

In the hippocampus, we observed no differences in GFAP reactivity between WT and 3xTg-AD mice. The percentage of the hippocampal area stained for the astrocyte marker GFAP did not significantly reduce after treatment with palm^11^-PrRP31, Dpr^3^-ghrelin, or liraglutide (Fig. 5.P).

In the amygdala, the difference between 3xTg-AD (saline treated) and WT (saline treated) group was apparent because of the significant increase of GFAP in 3xTg-AD (saline treated) group (*p* = 0.04) compared to WT (saline treated) group (Fig. 5.Q). The percentage of the brain area stained for GFAP, a marker of astroglial injury, was significantly reduced after treatment with palm^11^-PrRP31 (*p* = 0.03) and liraglutide (*p* = 0.04). However, the effect of Dpr^3^-ghrelin did not lead to a significant decrease, as quantified by the percentage of the immunohistochemically stained area.

In the cortex, there was also a significant increase in GFAP reactivity between WT and 3xTg-AD mice (*p* = 0.04). However, the treatment with palm^11^-PrRP31, Dpr^3^-ghrelin or liraglutide did not reduced astrocytosis (Fig. 5.R).

### Inhibition of TLR4 Pathway by Stable Analogues of Anorexigenic and Orexigenic Peptides

**Fig. 6:**
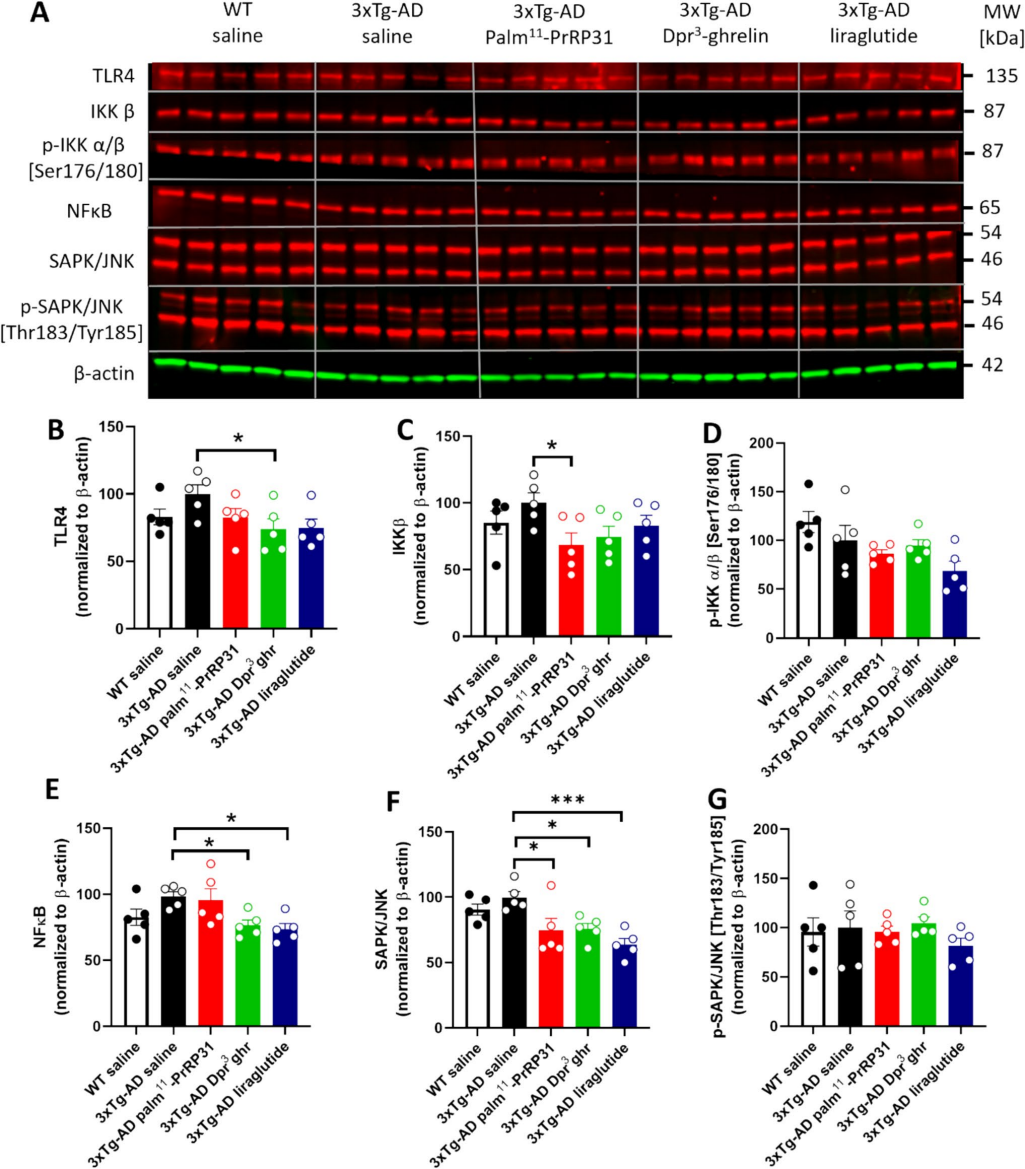
Inhibition of TLR-4 pathway. (**A**) Immunoblots of TLR-4 pathway in the hippocampi. Quantification of (**B**) TLR-4, (**C**) IKK β, (**D**) p-IKK α/β [Ser176/180], (**E**) NFκB, (**F**) SAPK/JNK, and (**G**) p-SAPK/JNK [Thr183/Tyr185]. Data are presented as the mean ± SEM. A one-way ANOVA with Dunnett’s test was used to analyze differences between groups. Significance levels are indicated as follows: *p < 0.05, and ****p < 0.0001 (*n* = 5 mice per group)

Activation of TLR4 pathway is connected to increased neuroinflammation. Consistently, we detected an increased level of TLR4 in the hippocampi of 3xTg-AD group (Fig. 6.B). Anorexigenic palm^11^-PrRP31 and liraglutide tended to decrease its level, and orexigenic Dpr^3^-ghrelin reduced the level significantly (*p* = 0.040) (Fig. 6.B). The slightly elevated IKK-β levels observed in 3xTg-AD mice treated with saline showed a tendency to decrease with all tested peptides. However, a significant reduction in the total level of IKK-β was only achieved with palm^11^-PrRP31 treatment (*p* = 0.018) (Fig. 6.C). The level of p-IKK α/β [Ser176/180] was not different between WT and 3xTg-AD mice, and no changes were observed after the treatment with anorexigenic and orexigenic peptides (Fig. 6.D). Subsequently, increased level of NFκB was observed in the hippocampi of saline-treated 3xTg-AD mice; both orexigenic Dpr^3^-ghrelin (*p* = 0.046) and anorexigenic liraglutide (*p* = 0.022) significantly decreased the level (Fig. 6.E). TLR-4 pathway is connected to increase in SAPK/JNK; the increased level in the hippocampi of 3xTg-AD mice was significantly lowered by all three tested peptides (Fig. 6.F, palm^11^-PrRP31, *p* = 0.018, Dpr^3^-ghrelin, *p* = 0.025 and liraglutide *p* = 0.0008), however, no effect on its phosphorylation was observed (Fig. 6.G).

### Attenuated Tau Phosphorylation after Treatment with Palm^11^-PrRP31 and Dpr^3^- Ghrelin

**Fig. 7:**
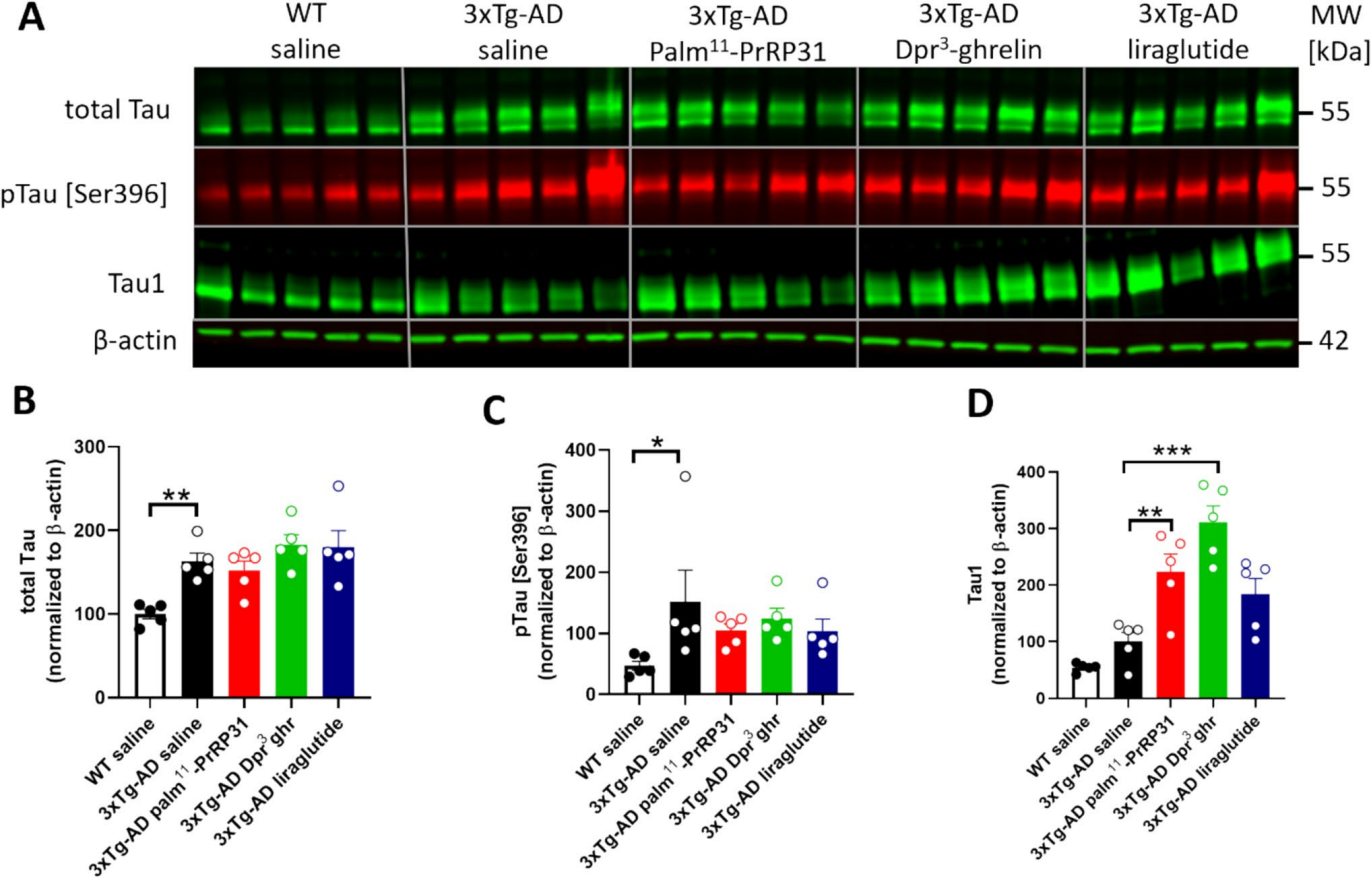
Reduction in hippocampal Tau phosphorylation after treatment with palm^11^-PrRP31 and Dpr^3^-ghrelin. (**A**) Immunoblots of Tau protein. Quantification of (**B**) total Tau (Tau1 antibody), (**C**) pTau [Ser396], and (**D**) nonphosphorylated Tau (Tau1 antibody) at serine epitopes 195, 198, 199, and 202. Data are presented as the mean ± SEM. A one-way ANOVA with Dunnett’s test was used to analyze differences between groups. Significance levels are indicated as follows: *p < 0.05, **p < 0.01, ****p < 0.0001 (*n* = 5 mice per group)

Tau protein levels were further determined in hippocampal lysates using WB (Fig. 7). Compared to the saline-treated WT group, the saline-treated 3xTg-AD group exhibited a significant increase in total Tau protein (*p* = 0.007), which was unaffected by treatment with palm^11^-PrRP31, Dpr^3^-ghrelin, or liraglutide (Fig. 7.B). Increase in hippocampal Tau phosphorylation was observed in 3xTg-AD mice, compared to WT animals, at [Ser396]; the phosphorylation then tended to attenuate after treatment with all tested compounds (Fig. 7.C). Oppositely, compared to saline treated 3xTg-AD mice, increased level of Tau1 after the treatment with palm^11^-PrRP31 (*p* = 0.007) and Dpr^3^-ghrelin (*p* < 0.001) was observed. Tau1 antibody recognized nonphosphorylated Tau at serine epitopes 195,198,199 and 202, therefore, the treatment with palm^11^-PrRP31 and Dpr^3^-ghrelin significantly attenuated Tau hyperphosphorylation. Treatment with liraglutide tended to increase Tau1 protein, albeit to a nonsignificant level (*p* = 0.09) (Fig. 7.D).

Double staining of 3xTg-AD mouse brain slices illustrates AT-100 (marker for pTau [Thr212 and Ser214]) and NeuroTrace™ (neuronal marker) in the hippocampal subarea cornu Ammonis 1 (CA1 area) (Suppl. Figure 2.). In the saline treated WT mice (Suppl. Figure 2.A-C) CA1 area the decreased signal of the pTau AT-100 marker is observed when compared to saline treated 3xTg-AD group (Suppl. Figure 2.D-F). Double staining of total Tau5 (visualized in red) and NeuroTrace™ (visualized in blue) in the CA1 area revealed the increased signal of Tau5 marker in the saline treated 3xTg-AD mice (Suppl. Figure 3.D-F) compared to their age-matched WT controls (Suppl. Figure 3.A-C).

**Fig. 8:**
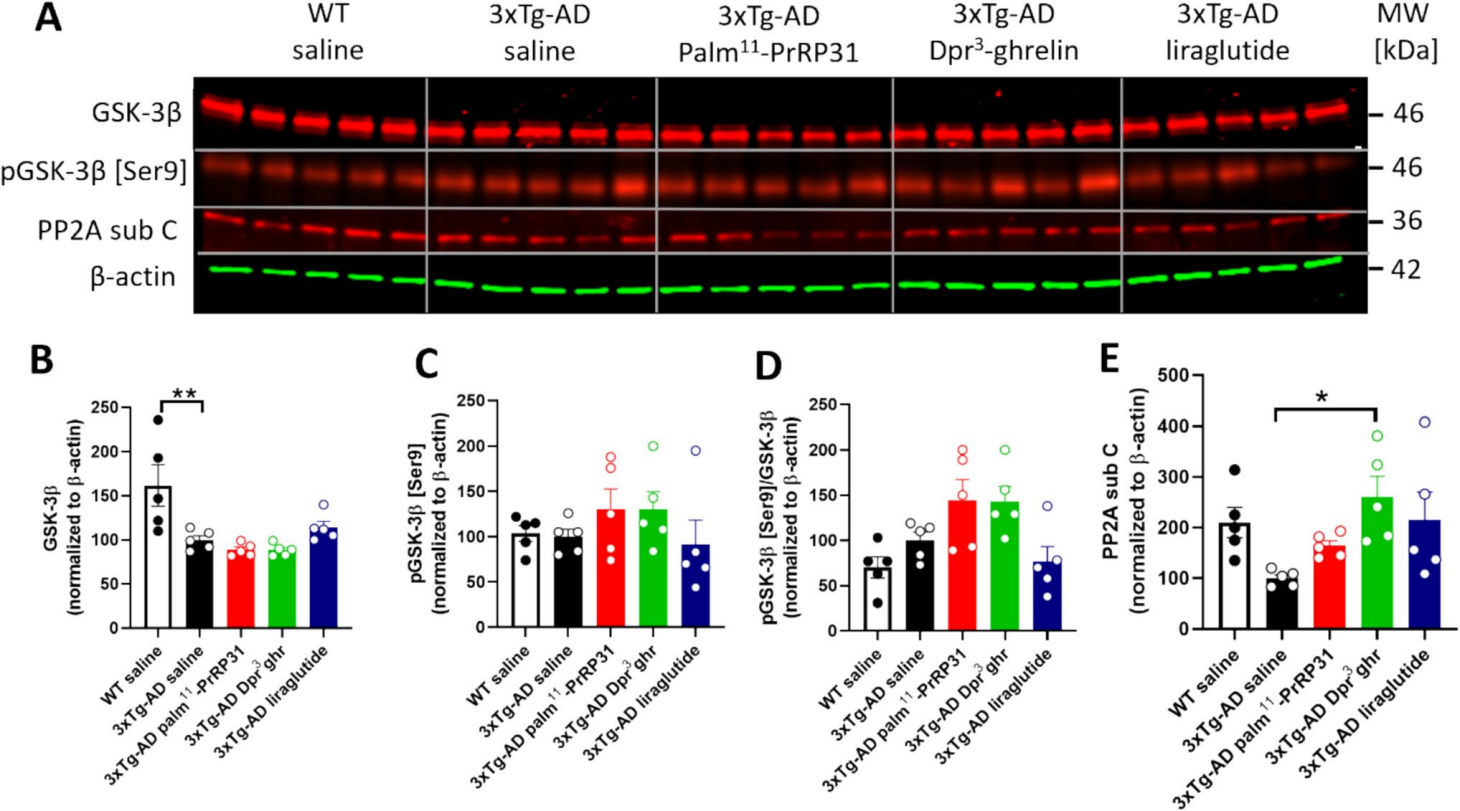
Levels of Tau kinase GSK-3β and Tau phosphatase PP2A in the hippocampi of 3xTg-AD mice. (**A**) Immunoblots of GSK-3β and PP2A. Quantification of (**B**) GSK-3β, (**C**) pGSK-3β [Ser9], (**D**) pGSK-3β [Ser9]/GSK-3β, and (**E**) PP2A subC. Data are presented as the mean ± SEM. A one-way ANOVA with Dunnett’s test was used to analyze differences between groups. Significance levels are indicated as follows: *p < 0.05, **p < *0.01* (*n* = 5 mice per group)

The level of hippocampal Tau phosphorylation in 3xTg-AD mice, and its subsequent attenuation after the treatment correspond to the level and activation of GSK-3β and PP2A, as the main Tau kinase and phosphatase, respectively (Fig. 8.). The level of GSK-3β was significantly decreased in hippocampi of 3xTg-AD mice (*p* = 0.004), compared to WT littermates. Although there were no differences in the phosphorylation of GSK-3β at inhibitory epitope [Ser9], the treatment with palm^11^-PrRP31 and Dpr^3^-ghrelin tended to increase its phosphorylation pointing to decreased kinase activity toward Tau protein. And conversely, PP2A subC level was decreased in the hippocampi of 3xTg-AD mice compared to WT mice, and the treatment with palm^11^-PrRP31 and liraglutide tended to increase its level, whereas the treatment with Dpr^3^-ghrelin significantly increased the level of PP2A subC (*p* = 0.011).

## Discussion

Alzheimer's disease, the most common form of neurodegenerative disease, is characterized by the accumulation of amyloid plaques and neurofibrillary tangles within the brain. These plaques consist primarily of Aβ deposits, while the tangles are composed of hyperphosphorylated Tau proteins. Additionally, AD is marked by brain inflammation, evidenced by heightened activity in the astrocytes and microglia, along with elevated levels of proinflammatory cytokines such as interleukin-1β, interleukin-6, and tumor necrosis factor-α. Animal models play a crucial role in the study of disease pathogenesis and potential therapeutic interventions. However, in the case of complex disorders like AD, the sheer variety of animal models poses unique challenges for researchers, complicating the development of effective treatments.

Obesity and its associated metabolic disorders, such as T2DM, were recently recognized as risk factors for AD development (Alford et al. 2018; Carvalho et al. 2013). Given this association, orexigenic and anorexigenic peptides, which regulate food intake in different ways, have been identified as potential neuroprotective agents. We previously highlighted the advantageous neuroprotective impacts of palm^11^-PrRP31 on spatial memory, synaptic plasticity, and Tau phosphorylation in THY-Tau22 mice, a model of AD-like Tau pathology (Popelová, Pražienková, et al. 2018a, b). Palm^11^-PrRP31 and liraglutide have been linked to reductions in Aβ plaque accumulation, along with decreased hippocampal microgliosis and cortical astrocytosis in APP/PS1 mice (Holubová et al. 2019). In a recent in vitro investigation employing a SH-SY5Y cellular model, Popelova et al. (Popelová et al. 2018a) illustrated that pre-treatment with the Dpr^3^-ghrelin analogue protected cells against methylglyoxal-induced toxicity and apoptosis.

Altogether, these findings indicate the potential therapeutic utility of this treatment approach for neurodegenerative conditions. Building on these results, we herein investigate the shared mechanisms behind the neuroprotective properties of two anorexigenic peptide analogues – palm^11^-PrRP31 and liraglutide – and Dpr^3^-ghrelin (a stable analogue of the orexigenic peptide ghrelin) using a 3xTg-AD model consisting of 10-month-old mice.

We found no significant changes in glucose, insulin, or leptin blood levels following four months of treatment with palm^11^-PrRP31, Dpr^3^-ghrelin, or liraglutide. We previously observed a similar effect of palm^11^-PrRP31 on APP/PS1 mice after two months of treatment with palm^11^-PrRP31 (Holubová et al. 2019). In the present study, the control group of WT mice (B6129SF2/J – 101,045) obtained from the Jackson Laboratory as a suitable WT control mice to 3xTg-AD mice, was significantly heavier than the 3xTg-AD mice, impeding a comprehensive metabolic evaluation.

Intraneuronal deposits of Aβ in the hippocampi and amygdalae of 3xTg-AD mice typically become evident at four months of age (Billings et al. 2005). The amygdala, another important subcortical region, plays a major role in the processing and memorizing of emotional reactions It is notably and consistently impacted by pathological alterations in AD ((Shira 2012); (Kacířová et al. 2021). At six months of age, extracellular deposits of Aβ appear in the hippocampus, amygdala, and cerebral cortex. At this point, intracellular accumulation is also detectable in the pyramidal neurons of the CA1 region in the hippocampus, as well as within the basolateral amygdala and cortical neurons (Billings et al. 2005). In our study, we observed a significant increase in intraneuronal deposits of Aβ (immunostained with 6E10 antibody) in the hippocampi, amygdalae, and cortices of 10-month-old 3xTg-AD mice compared to the saline-treated WT group which is in accordance with Muntsant et al. (2023) where they observed age-dependent progression of intraneuronal Aβ-positive cells in the prefrontal cortex, amygdala, dorsal and ventral hippocampus, and entorhinal cortex of 3xTg-AD mice (Muntsant et al. 2023). Although 3xTg-AD mice were 10 months old, they did not display immunoreactivity to extracellular deposits of Aβ (data not shown). We identified a significant overall reduction in intraneuronal Aβ plaques in the hippocampi and amygdalae of 3xTg-AD mice after treatment with all three analogues. For this study, we chose liraglutide as the comparative agent due to its neuroprotective properties against Aβ-induced spatial memory deficits and synaptic plasticity impairments in APP/PS1 mice (McClean & Holscher 2014; McClean et al. 2011).

According to the literature (Belfiore et al. 2019; Caruso et al. 2013), neuroinflammation in the 3xTg-AD mouse model becomes evident from seven months of age, characterized by astrocytosis and microgliosis. In agreement with other studies, we observed significantly increased immunoreactivity to Iba1 and GFAP in 10-month-old 3xTg-AD mice (Belfiore et al. 2019; Caruso et al. 2013). In line with the Aβ plaque load reduction, the neuroinflammatory response was reduced significantly after treatment with palm^11^-PrRP31, Dpr^3^-ghrelin, or liraglutide. Neuroinflammation, one of the hallmarks of AD, is a key contributor to neurodegenerative disorders (Bronzuoli et al. 2016). Activated microglia display distinct morphological changes characterized by reduced ramification, fewer branches, and a more pronounced hypertrophic state (amoeboid morphology) compared to non-activated microglia in a “resting” state (Sierra et al. 2016). Resting microglia play roles in Aβ clearance, surveillance of CNS parenchyma and neuronal activity, and neuroprotection during excitotoxicity. However, these safeguarding functions become impaired upon activation (McClean et al. 2011), after which activated microglia become involved in Aβ-induced synaptic loss, Tau pathology, and the release of inflammatory mediators that activate astrocytes (Hansen et al. 2018).

Neurodegenerative disorders manifest in sustained and gradual neuronal depletion, alongside an accumulation of cytotoxic substances such as extracellular debris, an increase in proinflammatory factors, and the generation of reactive oxygen species, which result in oxidative stress. These elements, along with the induction of factors capable of activating and attracting microglia, underscore the involvement of microglia in conditions like AD (Sierra et al. 2016). Although initially viewed as a transient phenomenon, microglial activation is now recognized as a significant and chronic aspect of disease progression. Studies have demonstrated that microglia sustain chronic activation in a process known as reactive microgliosis. A well-established characteristic of AD, reactive microgliosis is defined as microglial activation triggered by neuronal injury and perpetuated by subsequent microglial activation and neurotoxicity. This sets off a self-perpetuating cycle of microglial activation and neuronal damage, leading to progressive deterioration (Lull & Block 2010).

Astrocytes play a pivotal role in modulating the brain’s inflammatory response and the development of reactive astrogliosis, a phenomenon characterized by cellular enlargement and elevated expression of GFAP. Evidence from post-mortem analysis of tissues from AD patients and mouse models consistently reveals astrogliosis. Additionally, the severity of astrogliosis correlates with the extent of cognitive decline (Beach & McGeer 1988; Kashon et al. 2004; Mrak et al. 1996). Reactive astrocytes lose their neurosupportive functions, making neurons susceptible to excitotoxicity, metabolic, and oxidative stresses (Steele & Robinson 2012). Moreover, it has been suggested that reactive astrocytes may even promote further Aβ formation (Frost & Li 2017). In our study, treatment with palm^11^-PrRP31 and Dpr^3^-ghrelin markedly reduced microgliosis in the hippocampi, amygdalae, and cortices of 3xTg-AD mice. Palm^11^-PrRP31 and liraglutide significantly decreased astrocytosis in the amygdala. Such a reduction in chronic inflammation has been demonstrated to have neuroprotective effects (Tai et al. 2018).

Both reactive astrocytes and microglia are associated with activation of TLR4 pathway, which activates NFκB and further could accelerates neurodegeneration (Calvo-Rodriguez et al. 2020; Kumar 2019; Sun et al. 2022), and the therapeutics decreasing the TLR4 activation were suggested as potential neuroprotective compounds (Wu et al. 2022). Both anorexigenic and orexigenic compounds tested in our study tended to decrease the level of TLR4 in the hippocampi of 3xTg-AD mice. Activation of NFκB is mediated by activation of IKK complex (Wu et al. 2022). We observed reduced level of IKKβ, the catalytic subunit of the complex important for inhibition of NFκB (Li et al. 1999), after the treatment with our compounds, as well as tendency toward decreased level of p-IKKα/β. In concordance with the reduction in TLR4 and IKKβ levels, a decrease in NFκB was also observed, suggesting potential anti-inflammatory effects of the anorexigenic and orexigenic compounds we tested. Apart from NFκB activation, TLR4 is also connected to activation of SAPK/JNK which further promote pro-inflammatory processes and cytokine release (Dallas & Widera 2021); all our stable analogues of anorexigenic and orexigenic peptides significantly reduced the level of JNK.

Tau alterations in the 3xTg-AD mouse model, which typically occur by 12 months of age, become noticeably visible in CA1 neurons of the hippocampus. Specifically, aggregates of conformationally altered and hyperphosphorylated Tau are detected within the hippocampus (Billings et al. 2005; Oddo et al. 2003). In our study, we observed a significant increase in total Tau protein (immunostained with the Tau5 antibody) in the hippocampal lysates of 10-month-old saline-treated 3xTg-AD mice compared to saline-treated WT mice. These results align with a study by Chen et al. (Chen et al. 2017), who found a similar elevation in the Tau5 protein in nine-month-old 3xTg-AD mice. In our study, treatment with palm^11^-PrRP31, Dpr^3^-ghrelin, or liraglutide did not significantly change the protein level of total Tau. This finding is also in agreement with Chen et al. (2017) who observed no significant changes in Tau5 levels after two months of liraglutide administration.

We observed an increase in site-specific Tau phosphorylation, especially pTau [Ser396], in our saline-treated 3xTg- AD group compared to the saline-treated WT group. Similarly, Chen et al. (Chen et al. 2017) documented only a mild reduction in pTau [Ser396] after treatment with liraglutide in 10-month-old 3xTg-AD mice, a trend that was mirrored after treatment with palm^11^-PrRP31 and Dpr^3^-ghrelin. However, we also observed significantly attenuated Tau hyperphosphorylation at serine epitopes 195, 198, 199 and 202 (immunostained with the Tau1 antibody) after treatment with palm^11^-PrRP31 and Dpr^3^-ghrelin. These findings are in accordance with an earlier study by the same group (Chen et al. 2010), who demonstrated that ghrelin reduces abnormal Tau phosphorylation at [Ser 199] via the PI3-K/Akt-GSK pathway in vitro (Chen et al. 2010).

A study by Popelová et al. found that Tau protein phosphorylation in THY-Tau22 mice was attenuated at [Thr231, Ser396, and Ser404] (epitopes linked to AD progression) after palm^11^-PrRP31 treatment (Popelová, Pražienková, et al. 2018a, b). The effect of our peptide analogues could be connected to slight increase of phosphorylation of GSK-3β at inhibitory epitope [Ser9], which is the main Tau kinase (Sayas & Avila 2021) and further by the increased levels of PP2A, which is the Tau phosphatase (Martin et al. 2013).

## Limitations and Future Considerations

The 3xTg-AD mouse model is often criticized for not being an ideal representation of AD pathology due to several reasons. One significant drawback of the 3xTg-AD model is the diminishing effect of mutations over time. As the mice age, the expression of transgenes tends to decrease, leading to reduced levels of Aβ production and deposition. This reduction in Aβ pathology may not accurately reflect the progressive nature of AD in humans, where Aβ accumulation continues to worsen over time. While the 3xTg-AD model exhibits Aβ pathology, it lacks robust Tau pathology, another hallmark of AD. Tau protein aggregates into neurofibrillary tangles, contributing to neuronal dysfunction and cognitive decline in AD. Overall, we observed differences between Tau hyperphosphorylation in the animals. To effectively assess the effect of our analogues on Tau hyperphosphorylation, using older animals (at least 12 months old) might be more suitable. Additionally, the WT control mice (B6129SF2/J – 101,045) obtained from the Jackson Laboratory (Bar Harbor, Maine, USA) were significantly heavier than the 3xTg-AD mice, hindering a complete metabolic evaluation.

## Conclusions

**Fig. 9:**
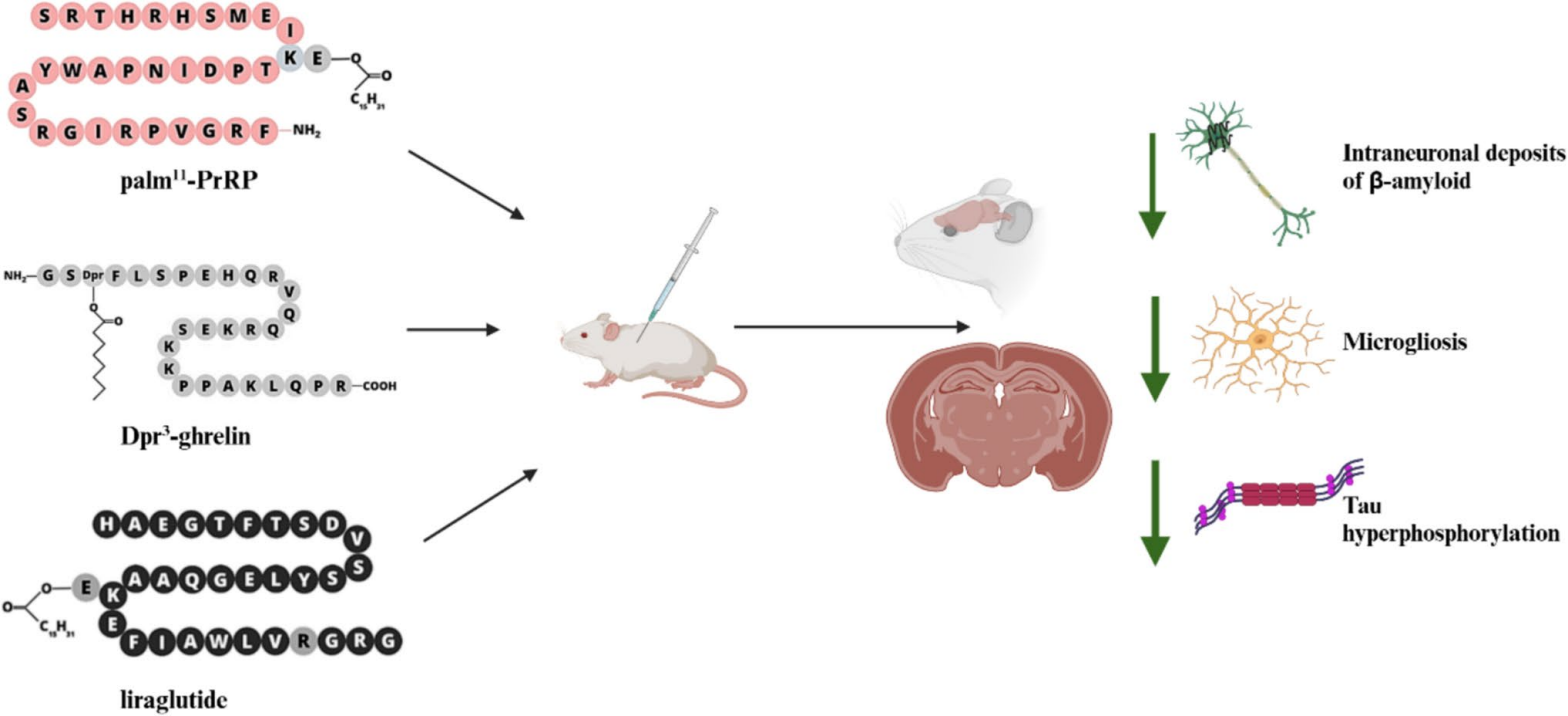
Reduction of microgliosis, intraneuronal Aβ deposits and tau pathology: a potential common mechanism used by both anorexigenic and orexigenic peptide analogues to improve AD pathology

In this study, we showed that stable lipidized peptide analogues of naturally occurring peptides involved in food intake regulation have the potential to lessen AD-like pathology in a 3xTg-AD mouse model. This finding applies to both anorexigenic (palm^11^-PrRP31, liraglutide) and orexigenic (Dpr^3^-ghrelin) compounds. Up to our best knowledge, this is the first study to investigate in a single mouse model with AD-like pathology the neuroprotective properties of both anorexigenic and orexigenic peptides. We have proven a significant improvement in AD pathology, underscoring the therapeutic potential of food-regulating peptides in neurodegenerative disorders. We hypothesize that the anti-inflammatory effect linked to inhibition of TLR4 pathway is a key mechanism associated with the reduction of AD-like pathology common to both orexigenic and anorexigenic peptides (Fig. 9).

## Supplementary Information

Below is the link to the electronic supplementary material.

Supplementary file1 (DOCX 3532 KB)

## Data Availability

The data could be obtained upon request to corresponding author.

## Abbreviations

**3xTg****-AD**: Triple transgenic mouse model of AD
**5xFAD**: Mouse model with familial form of AD
**Aβ**: Amyloid-beta
**AD**: Alzheimer's disease
**APP **: Amyloid precursor protein
**APP/PS1**: Mouse model with APPswe/PSEN1dE9 mutations
**APPSWE**: Swedish mutation in the APP
**BW**: Body weight
**CA1**: Region 1 of cornu ammonis
**CNS**: Central nervous system
**CRP**: C-reactive protein
**DAB**: 3,3′-Diaminobenzidine
**DG**: Dentate gyrus
**Dpr**^**3**^**-ghrelin**: Stable ghrelin analogue
**EDTA**: Ethylenediaminetetraacetic acidF
**ELISA**: Enzyme-Linked Immunosorbent Assay
**GFAP**: Glial fibrillary acidic protein
**GHS-R1a**: Ghrelin receptor 1a
**GLP-1**: Glucagon-like peptide 1
**GSK-3β**: Glycogensynthase kinase 3β
**Iba1**: Ionized calcium binding adaptor molecule 1
**IHC**: Immunohistochemistry
**IKKβ**: IκB kinase β
**mAb**: Monoclonal antibody
**NFκB**: Nuclear factor κB
**pAb**: Polyclonal antibody
**PBS**: Phosphate-buffered saline
**palm11-PrRP31**: Palmitoylated prolactin -releasing peptide analogue
**PP2A**: Protein phosphatase 2A
**PrRP**: Prolactin-releasing peptide
**PS1**: Presenilin 1
**PSEN1dE9**: Mutation in human presenilin 1 carrying the exon-9-deleted variant
**SAMP8**: The Senescence Accelerated Mouse-Prone 8
**SAPK/JNK**: Stress-activated protein kinase/Jun-amino-terminal kinase
**SC**: Subcutaneous(ly)
**SEM**: Standard error of the mean
**T2DM**: Type 2 diabetes mellitus
**Tau1**: Non-phosphorylated Tau protein at serine epitopes 195, 198, 199 and 202
**Tau5**: Total Tau protein
**Tau P301L**: Mutation at codon 301 of Tau protein (proline to leucine change)
**TLR-4**: Toll-like receptor 4
**TBS**: Tris-buffered saline
**WB**: Western blot
**WT**: Wild-type
