# Supplementary material for: Reduction of Neuroinflammation as a Common Mechanism of Action of Anorexigenic and Orexigenic Peptide Analogues in the Triple Transgenic Mouse Model of Alzheimer´s Disease
Source: J Neuroimmune Pharmacol. 2025 Feb 11;20(1):18. doi: 10.1007/s11481-025-10174-w (PMC11813825; doi:10.1007/s11481-025-10174-w)
Supplement: Supplementary file 1 — Supplementary file1 (DOCX 3532 KB) [file 11481_2025_10174_MOESM1_ESM.docx]

**Supplementary data**

**
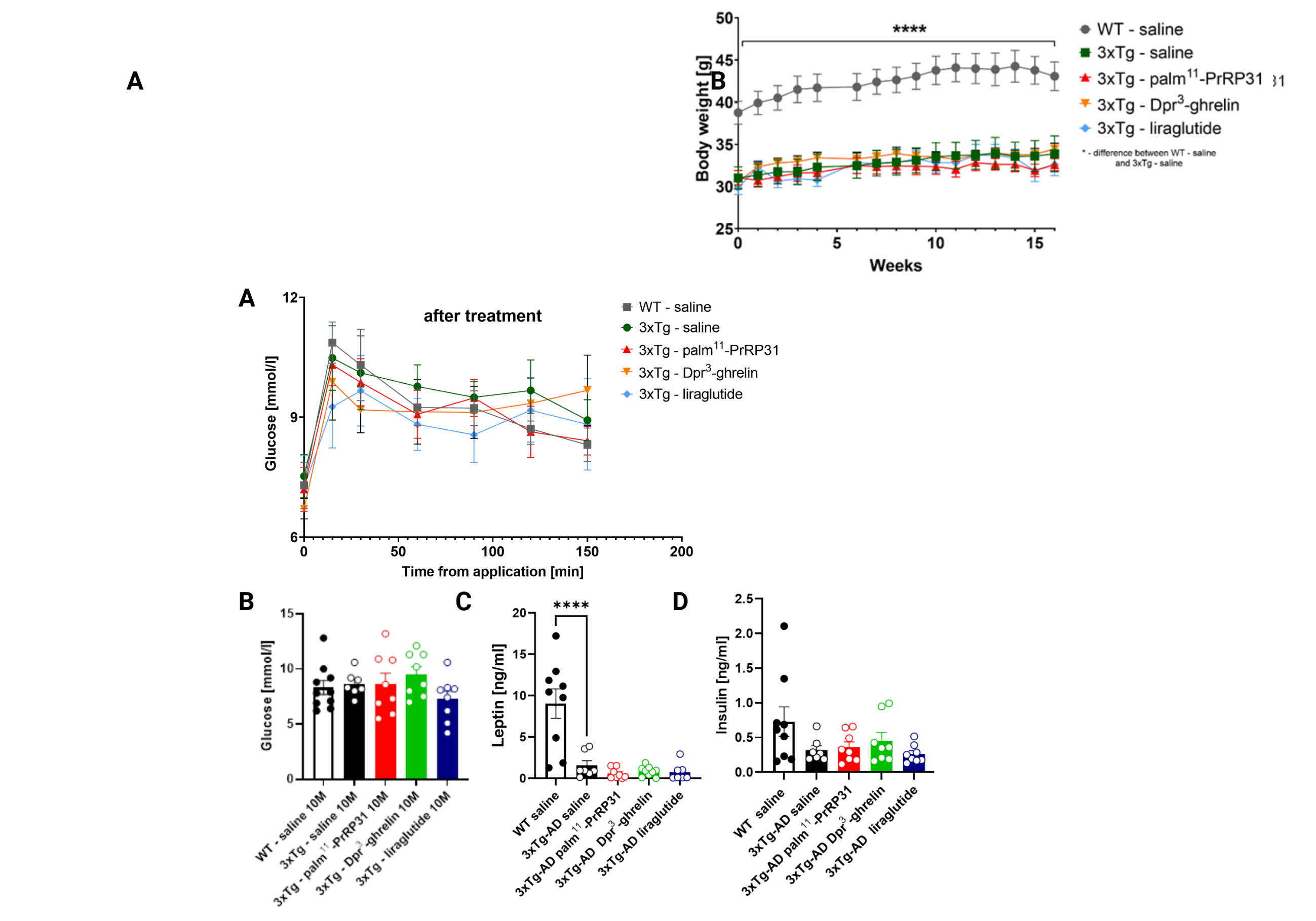
**

**Suppl. Fig.1. Oral glucose tolerance test (OGTT) and Metabolic parameters** – glucose (**B**), leptin (**C**), insulin (**D**), and OGTT (**A**) – measured at the end of the experiment. Data are presented as the mean ± SEM. A one-way ANOVA with Dunnett’s test was used to analyze differences between groups and by two-way ANOVA for OGTT. Significance levels are indicated as follows: **p*< 0.05, ***p*< 0.01, ****p*< 0.001, *****p*< 0.0001 (*n*= 8 or 9 mice per group).

**
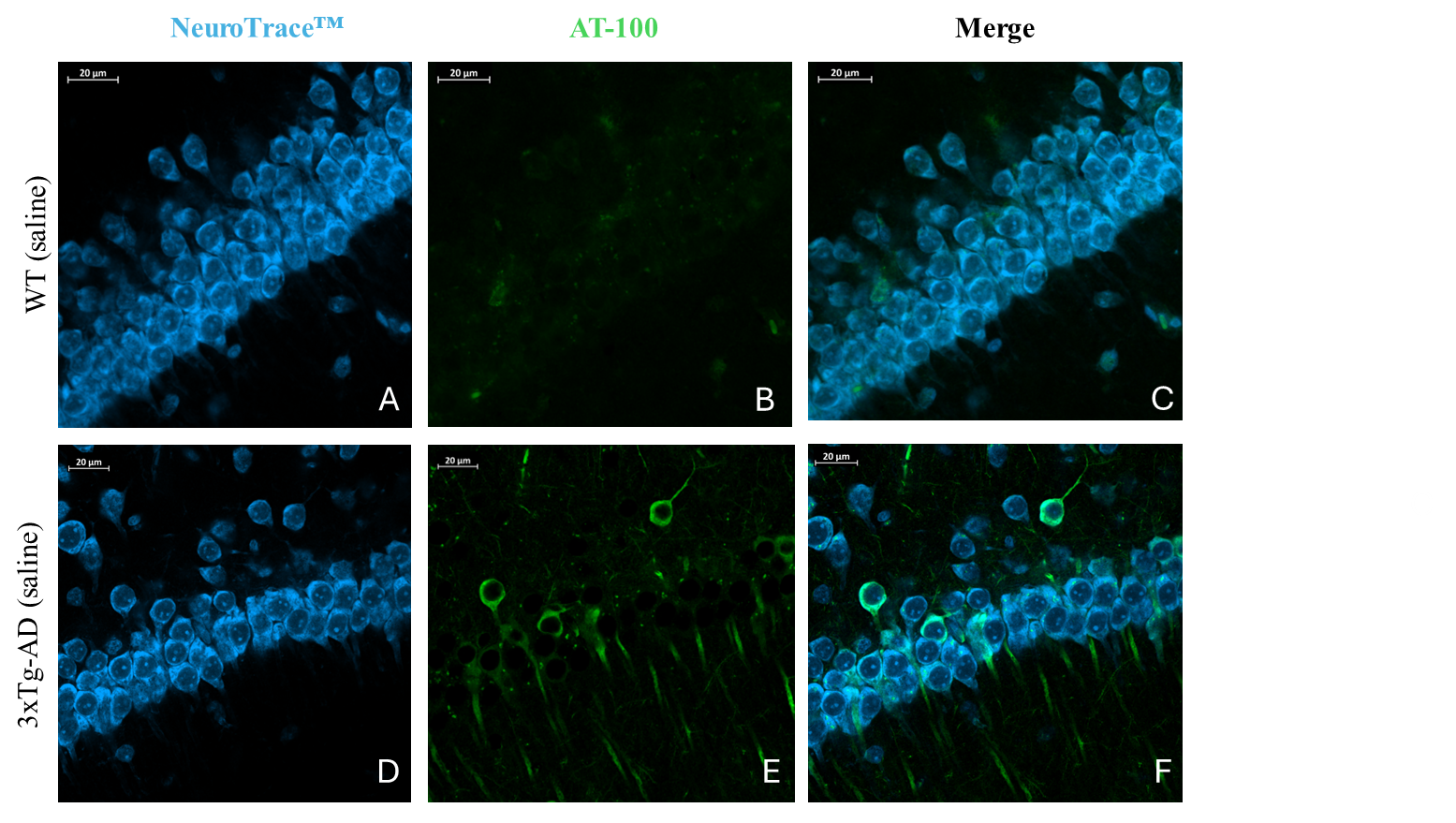
**

**Suppl. Fig.2.**Representative microscopic images of the CA1 region in (**D**–**F**) saline-treated 3xTg-AD mice and (**A**–**C**) age-matched WT controls. Images show immunohistochemical staining for **pTau AT-100** and **NeuroTrace™** at 40x magnification.

**
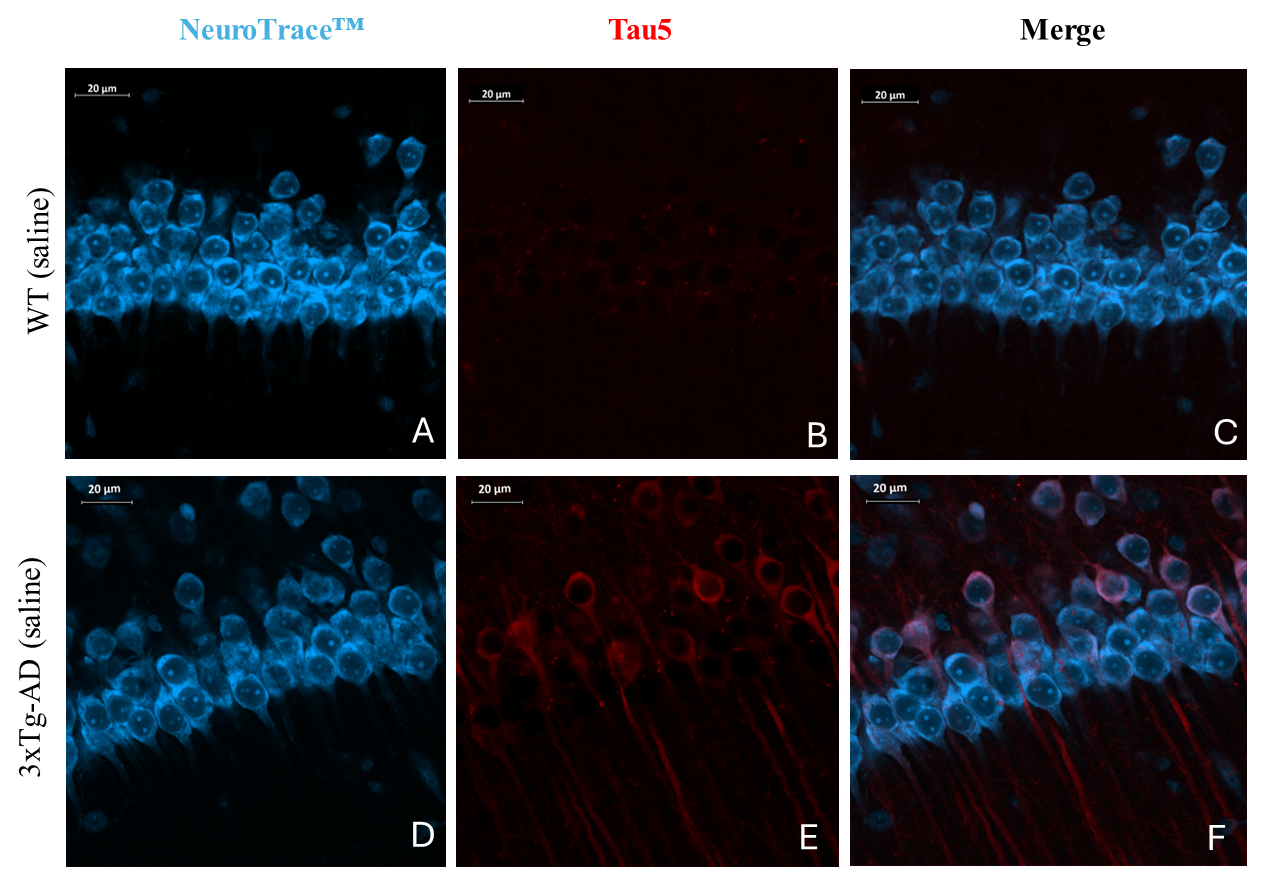
**

**Suppl. Fig.3.** Representative microscopic images of the CA1 region in (**D**–**F**) saline-treated 3xTg-AD mice and (**A**–**C**) age-matched WT controls. Images show immunohistochemical staining for total **Tau5** and **NeuroTrace™**. at 40x magnification.


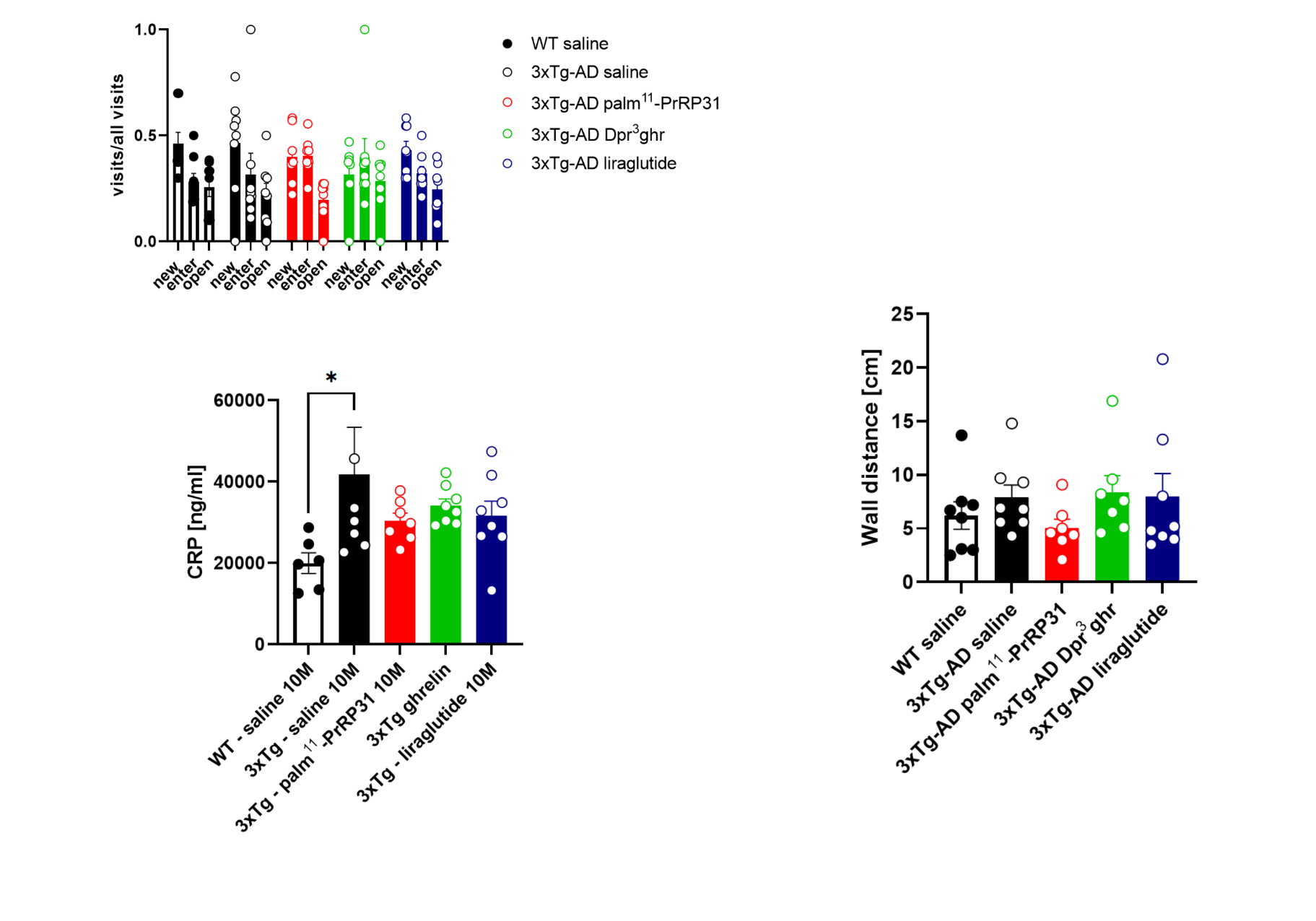


**Suppl. Fig.4.** Peripheral inflammation represented by C-reactive protein. Data are presented as the mean ± SEM. A one-way with Dunnett’s test was used to analyze differences between groups. Significance levels are indicated as follows: **p*< 0.05, *****p*< 0.0001 (n=6-8 mice per group).
